# Retina and Optic Disc Characteristics in Amblyopic and Non-amblyopic Eyes of Patients with Myopic or Hyperopic Anisometropia

**DOI:** 10.4274/tjo.54289

**Published:** 2017-01-17

**Authors:** Arzu Taşkıran Çömez, Elif Şanal Ulu, Yeliz Ekim

**Affiliations:** 1 Çanakkale Onsekiz Mart University Faculty of Medicine, Department of Ophthalmology, Çanakkale, Turkey

**Keywords:** Anisometropia, amblyopia, macula, Retinal nerve fiber layer, optical coherence tomography

## Abstract

**Objectives:**

To compare retinal and optic disc characteristics between amblyopic and non-amblyopic eyes in patients with myopic and hyperopic anisometropia measured with optical coherence tomography (OCT).

**Materials and Methods:**

Patients with myopic (25 patients: 17 female, 8 male; median age 27 years, range 16-40 years) and hyperopic (31 patients: 19 female, 12 male; median age 20 years, range 13-41 years) anisometropic amblyopia were included. Eye examination included determination of best-corrected visual acuity (BCVA) with a Snellen chart, measurement of manifest and cycloplegic refraction after pupillary dilation, alternate cover testing, globe movement evaluation, A-scan biometry for axial length, biomicroscopy, fundus examination, and OCT scanning. Main outcome measures were spherical equivalence, BCVA, axial length, retinal nerve fiber layer (RNFL) thickness, macular thickness, macular volume, and optic disc area.

**Results:**

In both myopic and hyperopic patients, the absolute value of the mean spherical equivalence was significantly greater in the amblyopic than non-amblyopic eyes, and the mean BCVA was significantly less in the amblyopic than the non-amblyopic eyes. In both myopic and hyperopic patients, there were no significant differences in mean RNFL thickness, macular thickness, macular volume, axial length, or optic disc area between amblyopic and non-amblyopic eyes.

**Conclusion:**

The amblyopic process may have no significant effect on the RNFL, macula, or optic disc. Further studies with more patients, including postmortem studies, may clarify the retinal, histopathologic, and anatomic differences between amblyopic and non-amblyopic eyes.

## INTRODUCTION

Amblyopia is a condition that includes a decrease in the best-corrected visual acuity (BCVA) without a known organic etiology.^1^ This condition develops most frequently in children aged ≤6 to 8 years and may affect one or both eyes.^1^ It is caused by the abnormal development of the visual cortex arising from several factors, including strabismus, blurred vision from refractive error, or visual deprivation.^2^ Although the visual cortex is the primary area responsible for amblyopia, changes in the retina and in the lateral geniculate body may also exist.^3,4,5,6,7^

Amblyopia is primarily a cortical disorder, caused by unequal competitive input from the two eyes into the primary visual cortex. Anisometropia may produce amblyopia via a loss of foveal resolution in the less-focused eye due to localized mechanisms of foveal inhibition with loss of stereo acuity and binocular function.^8^ Anisometropia is one of the leading causes of amblyopia, which is the only identifiable amblyogenic factor in 37% of cases.^9^ In a case-control sibling study, patients with anisometropia of at least 1 diopter (D) were shown to have a slight increase in the amblyopia or strabismus risk.^10,11^ Studies of normal human subjects have demonstrated that induced anisometropia greater than 1 D causes abnormalities in resolution and induces a suppression scotoma.^12^ In animal models of amblyopia caused by visual deprivation during the neonatal period, histologic changes have been noted in the lateral geniculate body and cortex.^3,4]3,4^ Similar observations have been reported in humans.^5,6^

Optical coherence tomography (OCT) is a non-invasive, non-contact device that measures retinal nerve fiber layer (RNFL) thickness, macular thickness, macular volume, and optic disc area.^13,14,15,16^ The RNFL thickness measured by OCT is similar to RNFL thickness measured histologically.^15^

The purpose of this study was to investigate differences in peripapillary RNFL thickness, macular thickness, macular volume, and optic disc area between the amblyopic and non-amblyopic eyes of patients with myopic or hyperopic anisometropia using OCT.

## MATERIALS AND METHODS

Patients with myopic (25 patients: 17 female, 8 male) or hyperopic (31 patients: 19 female, 12 male) anisometropic amblyopia were included in the study. All patients had no previous intraocular surgery, glaucoma, nystagmus, neurologic disease, or retinal disease. Exclusion criteria included strabismic and deprivation amblyopia. For all patients, amblyopia treatment had not been previously prescribed or implemented. Anisometropia was defined as an interocular difference in spherical equivalent refraction (spherical value + ½ cylinder value) ≥1 D and interocular difference in BCVA ≥2 lines of Snellen acuity.^17^ The study was approved by the Institutional Ethics Committee of Çanakkale Onsekiz Mart University. Written informed consent was obtained from each adult patient or from a parent or legal guardian of participants aged <18 years. The study was performed according to the guidelines of the Declaration of Helsinki for research involving human subjects. All patients had detailed eye examination that included BCVA determination with a Snellen chart (distance, 6 m); measurement of manifest and cycloplegic refraction after pupillary dilation (1% cyclopentolate hydrochloride and 1% tropicamide); alternate cover testing; extraocular movement testing; fundus examination; A-scan biometry for axial length; slit-lamp biomicroscopy; and OCT scanning (Opko/OTI Inc., Miami, FL, USA). Peripapillary RNFL thickness was measured using the fast RNFL thickness (3.4) scan protocol. The patients were asked to look at an internal fixation target and a circular scan with a diameter of 3.4 mm was centered around the optic disc. The location of the scan was observed to ensure the proper positioning in relation to the optic nerve head. The average of three consecutive OCT images of the RNFL was obtained.

Macular thickness was measured as the distance between the internal limiting membrane and retinal pigment epithelium using the fast macular thickness map protocol.^18^ Optic nerve head images were acquired with optic nerve topography scan mode.^19^

Data analysis was performed with statistical software (NCSS-2004, NCSS Inc., Kaysville, UT, USA). All quantitative variables were reported as mean ± standard deviation and range (minimum to maximum), and qualitative variables were expressed as a number (%). After assessing normality, mean values for the amblyopic and non-amblyopic eyes of both myopic and hyperopic groups were compared with t test or Mann-Whitney U test. Categorical variables between groups were compared with chi-square test. The association between amblyopia and retinal function was estimated by multivariate logistic regression analysis with hierarchical models and Pearson product moment correlation or Spearman rank correlation. All statistical analyses used 2-sided hypothesis tests. Statistical significance was defined as p≤0.05.

## RESULTS

Age was similar between patients with myopia (median, 27 years; range, 16 to 40 years) and those with hyperopia (median, 20 years; range, 13 to 41 years). In all patients, examination of the anterior segment, fundus, and intraocular pressure was normal. In both myopic and hyperopic patients, the absolute value of the mean spherical equivalence was significantly greater in the amblyopic than non-amblyopic eyes (p≤0.004), and the mean BCVA was significantly less in the amblyopic than the non-amblyopic eyes (p≤0.001, Table 1). In myopic patients, the cylindrical error was significantly greater in the amblyopic eyes (p≤0.001), whereas in hyperopic patients, the spherical error was significantly greater in the amblyopic eyes (p≤0.002, Table 1). In both myopic and hyperopic patients, there were no significant differences in mean RNFL thickness, macular thickness, macular volume, axial length, or optic disc area between amblyopic and non-amblyopic eyes (Table 1).

In the non-amblyopic eye of patients with myopia, patient age was negatively correlated with RNFL thickness, macular thickness, and macular volume, and BCVA was negatively correlated with axial length (p≤0.05, Table 2). In both amblyopic and non-amblyopic eyes of patients with myopia, significant correlations were noted between spherical equivalence and BCVA; spherical equivalence and axial length; macular thickness and macular volume; macular thickness and axial length; and macular volume and axial length (p≤0.05, Table 2).

In the amblyopic eyes of patients with hyperopia, spherical equivalence was negatively correlated with axial length, and optic disc area was positively correlated with axial length (p≤0.05, Table 2). In both amblyopic and non-amblyopic eyes of patients with hyperopia, significant correlations were noted between spherical equivalence and BCVA; and macular thickness and macular volume (p≤0.007, Table 2). No other significant correlations were noted between ocular parameters in patients with myopia or hyperopia.

## DISCUSSION

In the present study, there were no significant differences in peripapillary RNFL thickness, macular thickness, macular volume, axial length, or optic disc area between the amblyopic and non-amblyopic eyes of patients with myopia or hyperopia (Table 1). Numerous previous studies have evaluated the involvement of visual pathways, including parameters such as RNFL thickness and macular thickness in amblyopia in animals and humans; however, there are significant methodological differences such as measuring devices used, population age, subgroups of amblyopia, and refractive status. Lack of standard methodological approach makes comparison difficult and especially contributes to diverse and conflicting results.^7,16,17,20,21,22,23,24,25,26,27,28,29^

In a study by Walker et al.,^24^ RNFL measurements by OCT in 30 patients older than 18 years of age with amblyopia were performed. They did not find a difference in peripapillary RNFL or macular thickness between the amblyopic eye and fellow eye. Repka et al.^25^ performed peripapillary RNFL thickness of amblyopic and fellow eyes in 37 patients 7 to 12 years of age. They did not indicate that peripapillary RNFL thickness is thinner in eyes with moderate amblyopia compared with their fellow eyes. In a study by Kee et al.,^26^ OCT was performed on 26 children with unilateral amblyopia that was due to anisometropia or strabismus. OCT was also performed on 42 normal children. There were no differences in the fovea and the RNFL thickness found between normal children and children with amblyopia. There are two more studies that evaluated RNFL in amblyopic eyes. Neither of them found any differences between normal and amblyopic eyes.^27,28^ On the other hand, there are three studies conducted using OCT that suggest RNFL thickness may be greater in eyes with refractive amblyopia.^1,30,31^ In one of these studies, Yen et al.^30^ evaluated 38 patients with unilateral amblyopia. Among them, 20 patients had amblyopia with strabismus and 18 had refractive amblyopia without strabismus. RNFL was measured by OCT with scan pattern nerve head 2.0R (Carl Zeiss Meditec, Dublin, CA, USA). Average RNFL thickness was multiplied with their corresponding scan circumferences to estimate the integral values of the total RNFL area RNFL thickness (estimated integrals). In all 38 patients with unilateral amblyopia, the differences between the amblyopic eyes and the normal fellow eyes in RNFL thickness and in RNFL thickness (estimated integrals) were statistically significant. Another study included children younger (mean age, 7.7 years; range, 5 to 12 years) than the present patients, which may limit comparisons with the present data.^1^ Taken together, different age groups, different OCT devices, and different inclusion criteria (including strabismic patients, etc.) make healthy comparisons between the studies mentioned above and the present study almost impossible.^30,31^ In the current study, there was no significant difference in mean macular thickness between the amblyopic and non-amblyopic eyes in patients with myopia or hyperopia (Table 1). Previous studies reported conflicting results of macular thickness measurements from OCT in eyes with strabismic and anisometropic amblyopia.^1,20,21,24,26,30^ In a study by Kee et al.,^26^ there were no differences in the fovea thickness found between normal children and children with amblyopia. Other previous studies including patients with amblyopia and normal controls showed that macular thickness, foveal volume, and foveal thickness were similar in both eyes of the amblyopic group and were also similar to those eyes of the normal control groups.^26,32^

Kantarci et al.^33^ compared choroidal thickness and central macular and peripapillary RNFL thickness in adults with anisometropic amblyopia and also failed to find a difference in RNFL and central macular thicknesses, in agreement with our findings.

The present study showed no significant difference in macular thickness between amblyopic and non-amblyopic eyes in patients with myopia or hyperopia (Table 1). In contrast, a previous study in young myopic anisometropic amblyopic patients (mean age, 9.6 years; range, 5 to 18 years) showed thicker fovea and thinner inner and outer macular thickness in amblyopic eyes compared to normal eyes.^34^ Another study using OCT showed that central macular thickness was significantly increased in patients with anisometropic amblyopia, but mean RNFL thickness was similar between amblyopic (95.4 μm) and non-amblyopic eyes (94.0 μm).^35^ We excluded anisoastigmatism patients and included only spherical anisometropia patients. We did not find a significant correlation between axial length, macular thickness/volume or spherical equivalence. Anisometropic amblyopic eyes have statistically and clinically significant differences in refractive error. This refractive error can be attributed to corneal curvature changes, lens changes, anterior chamber depth and vitreous depth changes. In subjects with anisometropic amblyopia, interocular differences in spherical refractive error might be attributed to axial length with no differences in corneal curvature, whereas anisoastigmatism can also be observed, which results from asymmetric corneal curvature without a significant change in axial length.

In our study, there was no difference in spherical error between the study groups. However, cylindrical error was significantly different between groups. This finding means that the difference in mean spherical equivalent between groups is mainly caused by astigmatism. In other words, many of our subjects have anisoastigmatism, which does not have a significant effect on axial length. From this point of view, although amblyopic eyes have a longer axial length, this difference failed to reach statistical significance. We believe that including more subjects without significant anisoastigmatism may lead to statistically significant difference in axial length.

Optic disc area is directly associated with the number of nerve fibers in the optic nerve.^36^ The present study failed to find a significant difference between amblyopic and fellow eyes, as mean optic disc area was similar between amblyopic and non-amblyopic eyes of patients with both myopia and hyperopia (Table 1). Eyes with long diameters may have a large retinal surface and large optic disc.^37^ Conversely, small hyperopic eyes may have smaller optic discs. A deficiency of nerve fibers may be responsible for decreased visual acuity in amblyopic eyes.^38,39,40^

Other studies have shown that eyes with amblyopia may have smaller optic disc area than non-amblyopic eyes and healthy control eyes, and subclinical optic disc anomalies may be associated with amblyopia.^30,41^ However, the association between amblyopia and disc anomalies is controversial; the previously reported small disc area associated with amblyopia may have been caused by a correlation with hyperopia and anisometropia, and not necessarily because of a direct causal association between small disc area and amblyopia.^42^ The results of the previous studies are conflicting due to the differences in study design, OCT devices, and the subjects’ race, age, and amblyopia types. The majority of the studies included pediatric patients, whereas our study examined patients over 13 years of age, for whom amblyopia can no longer be treated. We believe that examining patients over 13 years old and comparing the retina characteristics in both myopic and hyperopic anisometropic patients makes an important contribution to the literature.

### Study Limitations

There are several limitations to our study. The small number of patients limits the power of the study, but the number of participants in this study is similar to other studies. The lack of a control group of normal children is another limitation, but we were able to use the non-amblyopic eye in each patient as a control.

## CONCLUSION

The present study showed no significant difference in mean RNFL thickness, macular thickness, macular volume, or optic disc area between amblyopic and non-amblyopic eyes in myopic and hyperopic anisometropic patients. This suggests that the amblyopic process may have no significant effect on the RNFL, macula, or optic disc in patients.

### Ethics

Ethics Committee Approval: Çanakkale Onsekiz Mart University Ethics Committee, Informed Consent: It was taken.

Peer-review: Externally peer-reviewed.

## Figures and Tables

**Table 1:**
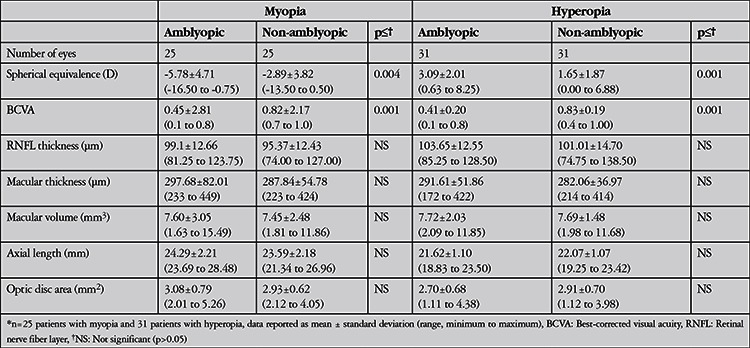
Ocular characteristics of amblyopic and non-amblyopic eyes in patients with myopia and hyperopia

**Table 2:**
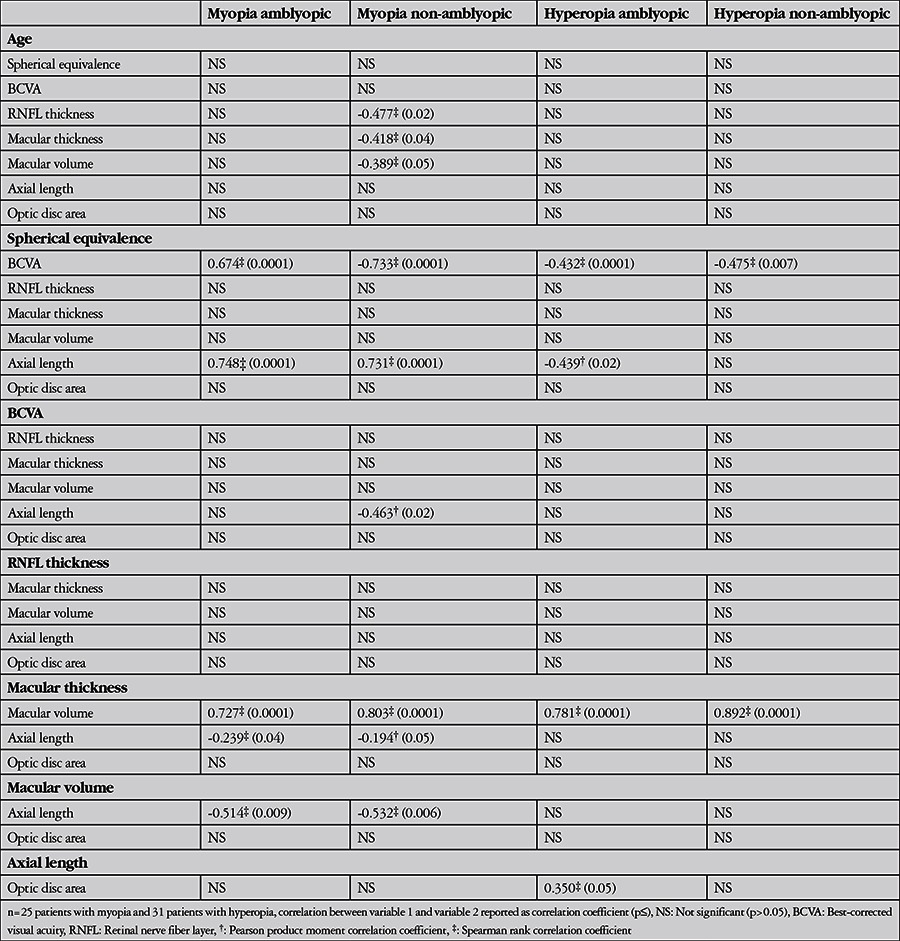
Correlations between ocular parameters in patients with myopia or hyperopia
